# Sick Headache

**Published:** 1882-11

**Authors:** 


### SICK HEADACHE.

Surgeon Major Roehring, of Amberg, reports, in No. 32 of the Allg. Med. Centr. Zeit., April 22d, 1882, a case of headache of long standing, which he cured by salicylate of sodium, which confirms the observations of Dr. Oehlschlager, of Dantzig, who first contended that we possessed in salicylic acid one of the most reliable remedies for neuralgia. This cannot astonish us if we remember that the action of salicylic acid is, in more than one respect, and especially in its influence on the nervous centres, analogous to quinine.

While out with the troops on manœuvre, Dr. Roehring was called to visit the sixteen-years-old son of a poor peasant family, in a neighboring village. The boy, who gave all evidences of living under bad hygienic surroundings, but who had shown himself very diligent at school, had been suffering, from his sixth year, several days every week, from the most intense headache, which had not been relieved by any of the many remedies tried for this purpose. A careful examination did not reveal any organic lesion or any cause for the pain, which seemed to be neuralgic in character, a purely nervous headache. Roehring had just been reading the observations of Oehlschläger, and knowing from the names of the physicians who had been already attending the poor boy that all the common remedies for neuralgia had been given a fair trial, thought this a good opportunity to test the virtue of salicylate of sodium. He gave the boy, who, in consequence of the severity of the pain, was not able to leave his bed, ten grains of the remedy every three hours, and was surprised to see the patient next day in his tent and with smiling face. The boy admitted that he for years had not been feeling so well as he did then. The remedy was continued, but in less frequent doses, for a few days longer; the headache did not return. Several months later Dr. Roehring wrote to the school teacher of the boy, and was informed that the latter had, during all this time, been totally free of his former pain, that he was much brighter than formerly, and evidently enjoying the best of health.

It may be worth while to give the remedy a more extensive trial, and the more so, as we are only too often at a loss what to do in stubborn cases of so-called nervous headache.—*The Medical and Surgical Reporter*, 1882.

